# Effects of Dietary Steroid Saponins on Growth Performance, Serum and Liver Glucose, Lipid Metabolism and Immune Molecules of Hybrid Groupers (♀*Epinephelus fuscoguttatus* × ♂*Epinephelus lanceolatu*) Fed High-Lipid Diets

**DOI:** 10.3390/metabo13020305

**Published:** 2023-02-19

**Authors:** Hongjin Deng, Jiacheng Zhang, Qihui Yang, Xiaohui Dong, Shuang Zhang, Weixing Liang, Beiping Tan, Shuyan Chi

**Affiliations:** 1 Laboratory of Aquatic Nutrition and Feed, College of Fisheries, Guangdong Ocean University, Zhanjiang 524088, China; 2 Guangdong Engineering Technology Research Center of Aquatic Animals Precision Nutrition and High Efficiency Feed, Zhanjiang 524088, China

**Keywords:** steroidal saponins, hybrid grouper, high-lipid diet, glucose and lipid metabolism, nonspecial immune

## Abstract

High-lipid diets are attributed to excessive lipid deposition and metabolic disturbances in fish. The aim of this experiment was to investigate the effects of steroidal saponins on growth performance, immune molecules and metabolism of glucose and lipids in hybrid groupers (initial weight 22.71 ± 0.12 g) fed high-lipid diets. steroidal saponins (0%, 0.1% and 0.2%) were added to the basal diet (crude lipid, 14%) to produce three experimental diets, designated S_0_, S_0.1_ and S_0.2_, respectively. After an 8-week feeding trial, no significant differences were found between the S_0_ and S_0.1_ groups in percent weight gain, specific growth rate, feed conversion ratio, protein efficiency ratio and protein deposition rate (*p* > 0.05). All those in the S_0.2_ group were significantly decreased (*p* < 0.05). Compared to the S_0_ group, fish in the S_0.1_ group had lower contents of serum triglyceride and low-density lipoprotein cholesterol and higher high-density lipoprotein cholesterol and glucose (*p* < 0.05). The activities of superoxide dismutase, catalase and glutathione peroxidase were significantly higher, and malondialdehyde contents were significantly lower in the S_0.1_ group than in the S_0_ group (*p* < 0.05). Hepatic triglyceride, total cholesterol and glycogen were significantly lower in the S_0.1_ group than in the S_0_ group (*p* < 0.05). Activities of lipoprotein lipase, total lipase, glucokinase and pyruvate kinase, and gene expression of lipoprotein lipase, triglyceride lipase and glucokinase, were significantly higher in the S_0.1_ group than in the S_0_ group. Interleukin-10 mRNA expression in the S_0.1_ group was significantly higher than that in the S_0_ group, while the expression of interleukin-6 and tumor necrosis factor-α genes were significantly lower than those in the S_0_ group. In summary, adding 0.1% steroidal saponins to a high-lipid diet not only promoted lipolysis in fish livers, but also activated glycolysis pathways, thus enhancing the utilization of the dietary energy of the groupers, as well as supporting the fish’s nonspecial immune-defense mechanism.

## 1. Introduction

Aquaculture has undeniably established its crucial role in global food security and nutrition. The total aquaculture production of the world has reached 122.6 million tons in 2020 [1]. The scale of aquaculture in the world is so large that the more aquatic feeds are required. In recent years, fish-meal and soybean-meal supply have been increasingly tight, which made the prices of some miscellaneous meal fluctuate from time to time, such as cotton meal, rapeseed meal and peanut meal, etc. [2,3]. In view of such a serious situation, full utilization of the energy effect of lipids and carbohydrates in feed to save protein is a more effective way. However, the higher dietary lipids or glucose easily led to the abnormal deposition of lipids in the liver and, directly affecting the health of fish, which would influence the fish yield and economic benefits [4].

Studies in Nile tilapia (*Oreochromis niloticus*), carp (*Cyprinus carpio*) and mice showed that saponins can decrease the total cholesterol (TC) and triglyceride (TG) contents and the activities of glutamic pyruvic transaminase (GPT) and glutamic oxalacetic transaminase (GOT) to protect the body [5,6,7,8]. Steroidal saponins, formed by the bonding of steroidal sapogenin and glycosides groups, were widely distributed in roots and rhizomes of plants, mainly found in monocotyledons such as agave, dioscoreaceae and genicaceae [9,10]. Structural diversity of steroidal saponins allows for a series of physiological activities, such as regulating the body’s metabolism of carbohydrates and lipids by increasing the activities of lipoprotein lipase and hepatic lipase [7,11]. The right amount of quillaja or soybean saponins have been proven to promote percent weight gain, protein efficiency ratio and protein deposition ratio in juvenile Japanese flounder (*Paralichthys olivaceus*) [12], sea bream (*Sparus aurata*) [13] and carp [14,15], meanwhile enhancing the immune defense by increasing the body’s anti-inflammatory and antioxidant capacities.

The grouper, as a common marine fish in China, had a production of 2.04 × 10^5^ tons in 2021, accounting for 11.09% of the marine carnivorous fish cultured in China [16]. However, when groupers consumed diets with higher lipids, the liver showed injury, including larger areas of lipid droplets in liver sections and lower enzyme activities of hepatic CAT and SOD [17,18]. An in vivo experiment of saponin on primary hepatocytes of hybrid groupers fed a high-fat diet showed that saponin had the ability to limit lipid accumulation and improve the oxidative stress in the liver. Groupers that were fed a diet containing 0.4% saikosaponin d displayed the best growth, and fish fed a diet containing 0.8% saikosaponin d had similar weight gain compared to the control group [19]. However, early studies showed that growth performance and enzyme activity of antioxidants were inhibited, and proinflammatory factors were upregulated in sea bream [13], carp [14,15] and juvenile turbot (*Scophthalmus maximus*) [20] fed saponins over 0.2%. Therefore, this experiment was designed to investigate the effects of different levels of steroidal saponins on growth performance, nonspecific immune molecules and glucose and lipid metabolism in hybrid groupers (♀*Epinephelus fuscoguttatus*×♂*E. lanceolatu*) fed high-lipid diets.

## 2. Materials and Methods

### 2.1. Experimental Diets

The formula of the basal diet is shown in Table 1. Steroidal saponins (Guangzhou Feixit Biotechnology Co., Ltd., Guangzhou, China) were added to the basal diet at 0%, 0.1% and 0.2% and form three isoproteic (52% crude protein) and isolipidic (14% crude lipid) diets, named as S_0_, S_0.1_ and S_0.2_. The ingredients were crushed (HNX-350, Beijing Huanya Tian yuan Machinery Technology Co., Ltd., Beijing, China) and passed through a 60-mesh screen. The solid materials were first mixed (B30 V-mixer, Lifeng Food Machinery Factory, Guangzhou, China) for 15 min, and then oil and water were added and mixed for 10 min to produce a moist dough. The dough was extruded by the twine-screw extruder (F-26, South China University of Technology, Guangzhou, China) through a 2.5 mm die. All pellet diets were air-dried at 25 °C and then stored at −20 °C until used.

### 2.2. Fish and Feeding Trial

The healthy hybrid groupers of consistent genetic background were purchased from a commercial hatchery (Hongxing Hatchery, Zhanjiang, China). The fish were then reared in an outdoor concrete pond (5 m × 4 m × 2 m), fed a commercial diet (50% crude protein, 8% crude lipid) and domesticated for two weeks. All fish were starved for 24 h, and then healthy groupers (initial weight 22.71 ± 0.12 g) were selected randomly and divided into 9 buckets (1 m_3_ Fiberglass farming buckets) with 25 fish each. The experiment was conducted in an indoor hydrostatic water culture system at the Marine Biological Research Base of Guangdong Ocean University (Donghai Island, Zhanjiang, China). The fish, reared in three groups with three replicates, were fed the experimental diets to apparent satiation at 8:00 and 16:00 daily for 8 weeks. During the feeding trial, fish were continuously oxygenated every day, kept at a temperature of 30.5 ± 0.8 °C, a salinity of 28–32, dissolved oxygen of 5–6 mg/L and an ammonia content of <0.2 mg/L. The culture water was replaced by 80% each other day.

### 2.3. Sample Collection and Chemical Analysis

At the end of the feeding trial, all fish in each bucket were made to fast for 24 h. In each replicate, fish were counted and weighed to calculate survival rate (SR), percent weight gain (PGR), specific growth rate (SGR), protein efficiency ratio (PER) and feed conversion ratio (FCR). Two fish were randomly selected in each replicate for whole-fish composition analysis. Three groupers were randomly selected from each replicate and measured for body length and weight, and their dissected livers and viscera were weighed to evaluate the condition factor (CF), hepatopancreas index (HSI) and viscerosomatic index (VSI).

Six fish were randomly selected from each replicate after weighing, and blood was collected from the tail vein. The blood samples were left at 4 °C for 12 h and centrifuged at 4 °C and 3500 rpm for 15 min. The serum obtained with centrifugation was for the analysis of biochemical indexes and antioxidative enzyme activities. The soybean-sized liver was cut in each replicate and washed with saline, and then preserved in 4% formaldehyde solution for making the histological section stained with PAS. Glycogen was quantified with software IPwin32 (6.0.0.260). Four livers in each replicate were obtained, two for measuring the activity of enzymes and another two for analyzing gene expression. The analysis methods for the index are listed in Table 2.

### 2.4. Quantitative RT-PCR Analysis of Gene Expression

Total RNA in the liver was extracted with Trizol reagent (Invitrogen, Carlsbad, CA, USA). The cDNA was synthesized by Prime Script RT kit (Takara, Osaka, Japan), and qRT-PCR was performed using SYBR Premix Ex Taq kit (Takara, Osaka, Japan) and carried out using a quantitative thermal cycler (Light Cycler480II, Roche Diagnostics, Basel, Switzerland). The reaction volume was 10 μL, containing 3.2 μL sterilized double-distilled water, 1 μL cDNA, 0.4 μL each primer and 5 μL SYBR Premix Ex Taq (Takara, Osaka, Japan). The cycle conditions were 30 s at 95 °C, then 35 cycles of 5 s at 95 °C, 25 s at 60 °C and 30 s at 72 °C. *β-actin* was as the reference gene to correct the results of different batches. The results were calculated using the 2^−ΔΔCt^ method in relative expression analysis. The primers used for qRT-PCR analysis are listed in Table 3.

### 2.5. Calculation and Statistical Analysis

Survival rate (SR, %) = 100 × final number of fish/initial number of fish.

Percent weight gain (PWG, %) = 100 × (final mean weight − initial mean weight)/initial mean weight.

Specific growth rate (SGR, %/d) = 100 × (final mean weight − initial mean weight)/number of rearing days.

Protein efficiency ratio (PER) = (final mean weight − initial mean weight)/(total feed intake × content of dietary protein).

Protein deposition rate (PDR, %) = 100 × (final mean weight × content of final body protein- initial mean weight × content of initial body protein)/(total feed intake × content of dietary protein).

Feed conversion ratio (FCR) = consumed feed weight/(final weight − initial weight + dead weight).

Feeding rate (FR, %BW/d) = 100 × consumed feed weight/[feeding days × (final fish weight + initial fish weight)/2].

Condition factor (CF, g/cm^3^) = 100 × body weight/body length^3^.

Hepatopancreas index (HSI, %) = 100 × liver weight/body weight.

Viscerosomatic index (VSI, %) = 100 × viscera weight/body weight.

The units of fish quantity, weight, length and experimental time were tail, g, cm and d, in that order.

All data were analyzed with a one-way analysis of variance (ANOVA) and significant differences among dietary groups were estimated by Tukey’s multiple comparison test. The results were expressed as means ± standard deviation (SD). Significant differences were chosen at *p* < 0.05. The images of the experimental results were drawn with Graph Pad Prism 8.0 software (8.0.2.263).

## 3. Results

### 3.1. Growth Performance

As indicated in Table 4, the FBW, PWG, PER, PDR and SGR of the fish in the S_0.1_ group were not significantly different from the S_0_ group and were significantly higher than those in S_0.2_ group (*p* < 0.05). The number of fish that died per replicate in group S_0_ was 0, 3 and 1, respectively; in group S_0.1_, it was 1, 1 and 1, respectively; and in group S_0.2_, it was 3, 3 and 2, respectively. The HSI and VSI of fish in the S_0_ group were not significantly different from the S_0.2_ group, but were significantly lower than those in the S_0.1_ group (*p* < 0.05).

### 3.2. Whole-Body Proximate Chemical Analysis

Body composition in the initial fish in each group was similar. After 8 weeks of a feeding trial, no significant difference was found in the moisture, crude protein and crude lipid levels of the whole fish among the experimental groups (*p* > 0.05) (Table 5).

### 3.3. Serum Biochemical Indexes

The content of TG in the S_0_ group was significantly higher than that in the S_0.1_ group, but significantly lower than that in the S_0.2_ group (*p* < 0.05). The content of serum TC and LDL-C was significantly lower in the S_0.1_ group than in the S_0.2_ group (*p* < 0.05). The level of HDL-C and GLU were significantly higher in the S_0.1_ and S_0.2_ groups than in the S_0_ group (*p* < 0.05) (Table 6).

### 3.4. Serum Antioxidative Index

The MDA contents in the S_0_ and S_0.2_ groups were significantly higher than that in the S_0.1_ group (*p* < 0.05) (Table 7). SOD activity of the S_0_ group was significantly lower than in the S_0.1_ group, but significantly higher than in the S_0.2_ group (*p* < 0.05). The CAT activity in the S_0.1_ and S_0.2_ groups were significantly higher than that in the S_0_ group (*p* < 0.05). Compared to the S_0.1_ group, GSH-PX activity in S_0_ and S_0.2_ groups was significantly decreased (*p* < 0.05).

### 3.5. Liver Histochemistry by PAS Stain

**Figure 1:**
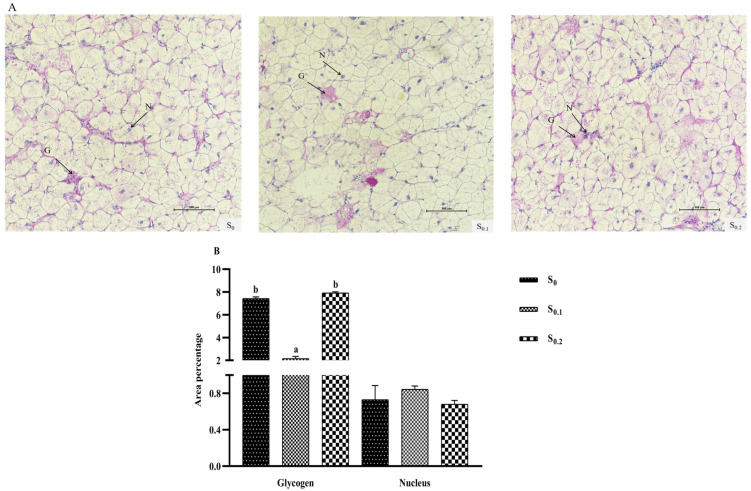
Effects of steroid saponin diets on the liver histochemistry of hybrid groupers after 8 weeks. Note: (**A**) Observation of liver sections (PAS staining, 200×). G, glycogen; N, nucleus. (**B**) Quantitative data on glycogen and nucleus. Different letters on the bars indicated significant difference (*p* < 0.05).

The results of the liver PAS stain are shown in Figure 1. The blue substance is the nucleus, and the purple substance is glycogen (Figure 1A). The percentage of nucleus in all groups was not significantly different (*p* > 0.05). The percentage of glycogen in the control group was significantly higher than in the S_0.1_ group, but was significantly lower than in the S_0.2_ group (*p* < 0.05) (Figure 1B).

### 3.6. Liver Biochemical Indexes

**Figure 2:**
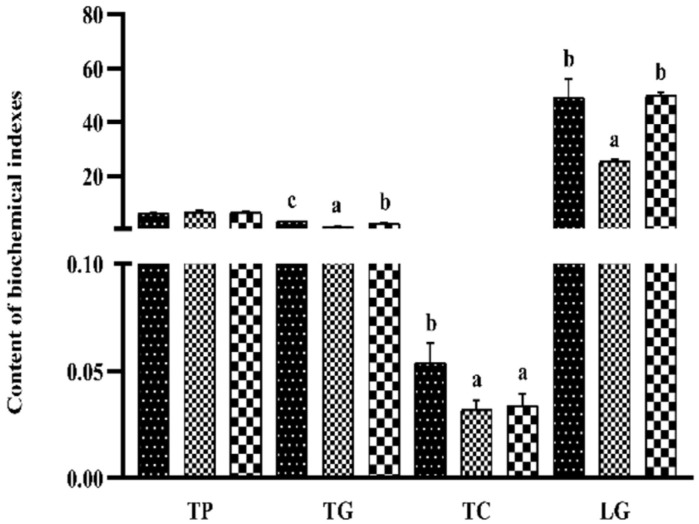
Effect of dietary steroid saponins on the biochemical indexes of fish livers. Note: (a–c): Values from smallest to largest. 3.7. Enzyme Activities and Genes Expression of Glucose and Lipid Metabolism in Liver.

There was no significant difference in hepatic TP among the three groups (*p* > 0.05) (Figure 2). Both the TG and TC contents of the S_0.1_ group were significantly lower than those of the S_0_ group (*p* < 0.05). The LG of the S_0_ group was significantly higher than that of the S_0.1_ group (*p* < 0.05) and was not significantly different from the S_0.2_ group (*p* > 0.05).

**Figure 3:**
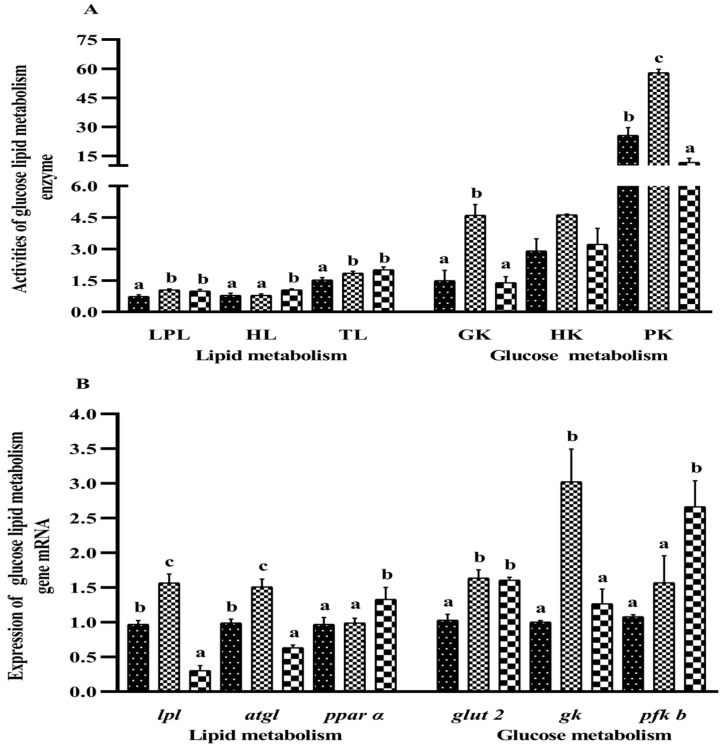
Effect of dietary steroidal saponins on glucose and lipid metabolism in the liver in hybrid groupers. Note: (**A**) enzyme activities of glucose and lipid metabolism; (**B**) gene expressions of glucose and lipid metabolism. (a–c): Values from smallest to largest.

From Figure 3A, enzyme activities of LPL and TL in the S_0.1_ and S_0.2_ groups were significantly higher than those in the S_0_ group (*p* < 0.05). Higher HL activity was found in the S_0.2_ group (*p* < 0.05), while it was not significantly different between the S_0_ and S_0.1_ groups (*p* > 0.05). The activities of GK and PK were significantly higher in the S_0.1_ group than in the other two groups (*p* < 0.05). No significant difference was found in the HK activity in all groups (*p* > 0.05).

The *lpl* and *atgl* mRNA expressions were significantly higher in the S_0.1_ group than in the other two groups (*p* < 0.05) (Figure 3B), while gene expression levels of *lpl* and *atgl* in the S_0.2_ group were significantly lower than those in the S_0_ group (*p* < 0.05). The *ppar α* mRNA expression did not show significant differences between the S_0_ and S_0.1_ groups (*p* > 0.05), while it was significantly higher in the S_0.2_ group (*p* < 0.05). The expression of *glut2* mRNA was significantly higher in the S_0.1_ and S_0.2_ groups than in the S_0_ group (*p* < 0.05). The *gk* mRNA expression was significantly lower in the S_0_ and S_0.2_ groups than in the S_0.1_ group (*p* < 0.05). The expression of *pfk b* mRNA was not significantly different between the S_0.1_ and S_0_ groups and was significantly lower than in the S_0.2_ group (*p* < 0.05).

### 3.7. Liver Immune Molecules

**Figure 4:**
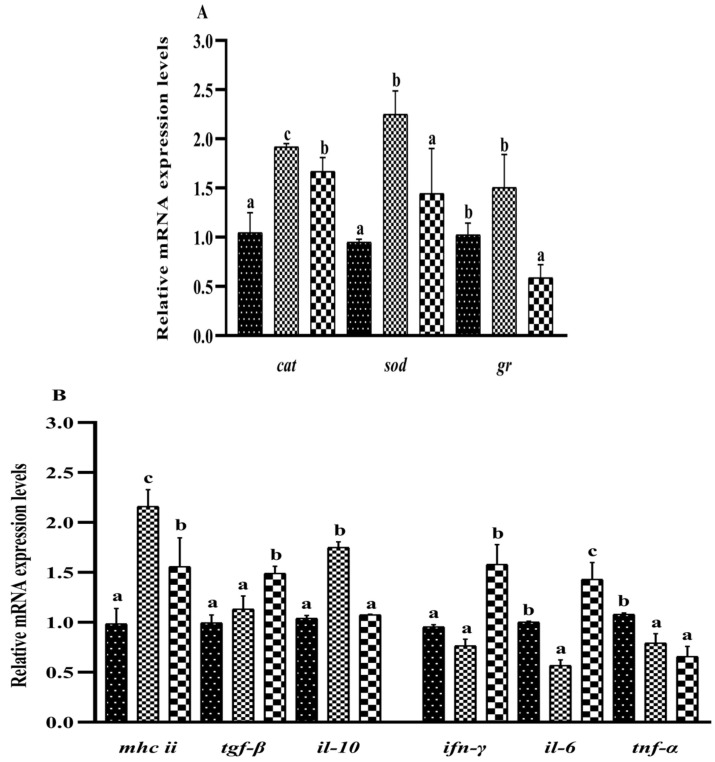
Effect of dietary steroidal saponins on the expression of genes related to immune molecules in hybrid groupers. Note: (**A**) genes of antioxidative enzyme; (**B**) genes of inflammatory factors. (a–c): Values from smallest to largest.

As Figure 4A shows, the mRNA expressions of the genes *cat* and *sod* were significantly higher in the S_0.1_ group than in the other two groups (*p* < 0.05). The mRNA expression of the *gr* was not significantly different between the S_0_ and S_0.1_ groups, but it was higher than in the S_0.2_ group (*p* < 0.05). The *mhc* II and *il*-10 mRNA expressions in the S_0.1_ group were significantly upregulated when compared with other groups (*p* < 0.05). The *tgf*-β mRNA expression in the S_0_ and S_0.1_ groups was lower than in the S_0.2_ group (*p* < 0.05). The expressions of *ifn*-γ and *il*-6 mRNA in S_0.2_ group were significantly higher than in other groups (*p* < 0.05). The *tnf*-α mRNA expression in the S_0.1_ and S_0.2_ groups were significantly lower than in the S_0_ group (*p* < 0.05).

## 4. Discussion

In most fish, dietary lipids are able to promote protein deposition efficiently in vivo, which is known as the protein-saving effect. Although the appropriate dietary lipid level for hybrid groupers was 10% [17,23,24], a higher lipid level in the diet was used in the practical feed in order to realize the protein-saving effect. However, excessive dietary lipids would cause lipid accumulation, inflammation and decreased PER and PDR in hybrid groupers, hybrid yellow catfish (*Pelteobagrus fulvidraco* × *P. vachelli*) and yellow croakers (*Larimichthys crocea*), inhibiting fish growth [25,26,27]. In this experiment, when 0.1% steroidal saponins was added to the diet with 14% crude lipids, more PER and PDR were obtained, but when steroidal saponins was up to 0.2%, fish growth was significantly inhibited. Studies in Atlantic salmon (*Salmo salar* L) [28] found that dietary addition of 0.2% soya-saponins increased fish PER and PDR, but supplementation of 0.2% or 0.32% soya-saponins decreased the weight-gain rate of carnivorous field eels (*Monopterus albus*) [29] and Japanese flounder [12]. Research in omnivorous carp [30] found that growth performance was significantly inhibited when dietary Momordica charantia saponins were above 0.64%. Thus, high doses of saponins were toxic to fish and decreased growth. Dietary soya-saponins over 0.25% significantly caused a decrease in the specific growth ratio and feed efficiency ratio in juvenile turbot (*Scophthalmus maximus*) [20]. However, these differences in the results might be related to saponin dose, species habits, feeding habits and the saponin type. The addition of 0.05–1% *Panax notoginseng* extract (with 80% saponins) to high-lipid diets (15%) promoted the growth of hybrid groupers. Although the growth was increased above 0.5% [31], in which the initial grouper size was larger than in the present study, we thought that the larger fish would have greater tolerance. However, the groupers of a similar size to those in the present study were fed a diet containing 0.4% saikosaponin d, which gave the best growth, and fish fed a diet containing 0.8% saikosaponin d had similar weight gain compared to the control group. Saikosaponin is a triterpenoid saponin. The saponin used in this experiment is a steroidal saponin extracted from mulberry leaves. The two types of saponin have different functions. Perhaps the type of the saponin made a difference in the dietary dose and effect on fish. The above research was combined to show that the addition of appropriate levels or type of saponins to high-lipid diets was helpful for playing a role in the conservation of protein and to increase PER and PDR, thus resulting in promoting fish growth.

Compared to terrestrial animals, aquaculture animals are characterized by a shorter digestive tract that limits the ability to digest and utilize food. High-lipid diets would increase the burden on fish and cause problems such as disorders of lipid metabolism in the serum and liver, thus affecting body health [32,33,34]. In a non-high-lipid diet, saponin reduced TC, TG and LDL-C and increase HDL-C in the serum of pacific white shrimp (*Litopenaeus vannamei*) [35] and juvenile turbot [36]. In the present experiment, the addition of steroidal saponins to the high-lipid diet significantly increased the enzyme activities of LPL and HL and upregulated the expression of *lpl*, *atgl* and *ppar α* genes in livers of hybrid groupers. Meanwhile, a decrease in TG and TC levels in serum and the liver of hybrid groupers was also observed, which is consistent with the trend of lipid-metabolizing enzymes in the liver. The saponin could activate the PPAR signaling pathway to upregulate the gene expression of *lpl*, *atgl* and *ppar α* to stabilize lipid metabolism disorders of hybrid groupers fed with a high-lipid diet [19]. It was indicated that steroidal saponins probably enhanced the hydrolysis of triglycerides to fatty acids through activating the PPAR signaling pathway, which subsequently activated the LDL and HDL receptor families in the cholesterol metabolic pathway to mediate cholesterol transport [18,37,38].

In addition, the intake of a high-lipid diet is often accompanied by disturbances in the glucose metabolism in fish, mainly in terms of wave blood glucose and hepatic glycogen content [33,34,39]. Saponins were found to significantly reduce hepatic glycogen and blood glucose levels and elevated the enzyme activities of glycolysis in carp that were fed with a high-starch diet [30]. In this experiment, the addition of steroidal saponins to the high-lipid diets significantly upregulated hepatic *GLUT2*, *GK* and *PFK b* gene expression, increased GK and PK enzyme activities, and decreased the hepatic glycogen content. When the organism was stimulated, *GK*, *HK* and *PFK b* expression in the AMPK signaling pathway was able to be activated, which subsequently regulated gene expression and glycolysis and provided energy to the organism [30,40]. The steroidal saponins might promote grouper growth by activating the expression of the *PFK b* gene in the AMPK signaling pathway and subsequently upregulating gene expression and the activity of enzymes in the glycolysis pathway. Dietary carbohydrates could be better energized by steroidal saponin stimulation, which was more conducive to the protein-sparing effect.

Saponins have been confirmed to reduce MDA content and enhance the antioxidant capacity of carp [6], white shrimp [41] and swimming crab (*Portunus trituberculatus*) [42], by increasing SOD, CAT, GSH-PX and GR activities and genes expression. In this study, enzyme activities and gene expression of SOD, CAT, GSH -PX and GR in groupers fed diets containing 0.1% steroidal saponins were significantly increased, and MDA levels in serum also decreased. At the same time, the expression of cell factors *mhc II*, *il-10* and *tgf-β* genes were upregulated, and *inf-γ*, *il-6* and *tnf-α* gene expressions were downregulated in groupers fed diets containing 0.1% steroidal saponins. In a similar trend, higher expressions of *mhc II*, *il-10* and *tgf-β* genes and lower expressions of *inf-γ*, *il-6* and *tnf-α* genes in the liver were found in turbot [43], sea bream [44] and carp [45,46] fed the diet with saponins. The expression of *mhc* II in the antigen presentation pathway might be upregulated by the saponins in groupers, which enables T cells to recognize antigens [47], together with the higher expression of *il-10* and *tgf-β* genes to exhibit the anti-inflammation in the body’s immune system. Saponins could act on the NF-κB signaling pathway to play a role in inflammation in the enterocytes [48] and also enhance the antioxidant capacity of carp [45] and mice [49] by acting on the Nrf2 signaling pathway and upregulating *sod*, *cat* and *gr* gene expressions, which then alleviate the liver damage. As a result, the steroidal saponins effectively improved the antioxidant ability and removed peroxide products, etc., in the body through the nonspecial immune factors maintaining the oxidative homeostasis, and thus enabled the orderly metabolism of nutrients in the body.

In conclusion, compared to the fish that were fed a diet without steroidal saponins, groupers that ingested a high-lipid diet with 0.1% steroidal saponins could achieve a better protein efficiency ratio, lower blood lipids and a healthier liver, which were all derived from raising the efficiency of the glycolipid metabolism for energy supply. Meanwhile, 0.1% steroidal saponins helped increase the nonspecial immune-defense mechanism of the fish by regulating immune molecules.

## Figures and Tables

**Table 1:** Ingredients (g/100 g diet) and proximate composition (% dry matter).

Ingredients	S_0_
Fish meal	36.00
Poultry by-product meal	10.50
Soybean meal	6.00
Concentrated cottonseed protein	19.00
Wheat flour	16.00
Fish oil	4.25
Soybean oil	4.25
Choline chloride	0.50
Ca (H_2_PO_4_)_2_	1.50
Vitamin C	0.05
Vitamin mix ^1^	0.50
Mineral mix ^1^	0.50
Betaine	0.50
Ethoxyquin	0.10
Steroid saponins	0.00
Microcrystalline cellulose	0.35
Total	100.00
Proximate analysis (%)	
Moisture	11.50
Crude protein	52.48
Crude lipid	13.93
Crude ash	11.74
Gross energy (KJ g^−1^ DM)	20.52

^1^ Vitamin premix and mineral premix were provided by Qingdao Master Biotech Co., Ltd., Qingdao, China.

**Table 2:** The chemical analysis used in the experiment.

Items	Methods	Item Code	Reference/Assay Kits/Section
**Composition of diets and whole body**			
Moisture	Drying at 105 °C to constant weight		Association of Official Analytical Chemists [21]
Crude lipid	Soxhlet extractor method (Petroleum ether)		
Crude ash	Combustion to a constant weight at 550 °C		
Crude protein	Dumas’s combustion method		Jean Baptiste Dumas (2018) [22]
**Serum biochemical indexes**			
Total cholesterol (TC, mmol/L)	Single reagent GPO-PAP method	A111-1-1	Assay kits (Nanjing Jiancheng Bioengineering Institute, Nanjing, China)
Triglyceride (TG, mmol/L)	Single reagent GPO-PAP method	A110-1-1	
Low-density lipoprotein cholesterol (LDL-C, mmol/L)	Microplate method	A113-1-1	
High-density lipoprotein cholesterol (HDL-C, mmol/L)	Microplate method	A112-+1-1	
Glucose (GLU, mmol/L)	GOD-PAP method	A154-1-1	
Malondialdehyde (MDA, nmol/mgprot)	TBA method	A003-1	
Superoxide dismutase (SOD, U/mL)	Hydroxylamine method	A001-2-2	
Catalase (CAT, U/mL)	Ammonium molybdate method	A007-1-1	
Glutathione peroxidase (GSH-PX, U/L)	Colorimetric method	A005-1-2	
**Hepatic enzyme activity**			
Total protein (TP, mgprot/mL)	Microplate method	A045-4-1	
Liver glycogen (LG, mg/g)	Colorimetric method	A043-1-1	
Total lipase U/mgprot)	Colorimetric method	A067-1	
Hepatic lipase (HL, U/mgprot)	Colorimetric method	A067-1-1	
Lipoprotein lipase (LPL, U/mgprot)	Colorimetric method	A067-1-2	
Glucokinase (GK, ng/mL)	Competition method, ELISA kit	H439-1	
Hexokinase (HK, nmol/min/mgprot)	Spectrophotometric method	A077-3	
Pyruvate kinase (PK, U/mgprot)	Ultraviolet colorimetric method	A076-1-1	
**Histochemistry observation**			
Liver Section	Periodic Acid Schiff (PAS)		Made by Wuhan Service Biotechnology Co, China.

**Table 3:** Primers pair sequences for qRT-PCR.

Target Gene	Nucleotide Sequence (5′-3′)	Accession No.
Lipoprotein lipase (*lpl*)	F: CCACCTGTTCATCGACTCCC R: TCGGACGGACCTTGTTGAT	EU683732.1
Adipose triglyceride lipase (*atgl*)	F: GAGGACAATAAAGGCGGTGAG R: AGCTTTGTGCAGGGTGGGT	KY649281.1
Peroxisome alpha (*ppar α*)	F: TGCTCGCCTCCAGTATGAA R: GTCCAGCTCCAGCGTGTTA	FJ196234.1
Glucose transporter protein 2 (*glut 2*)	F: TGTTCTGCTTTTCGGCTTC R: CAGTTCCGCATTGTCTATG	KY656467
Glucokinase (*gk*)	F: TGGGTTTTACCTTCTCCTT R: AGTCCCCTCGTCTCTTGAT	MH213270
Phosphofructokinase type b (*pfk b*)	F: AAACGCCCATGCAAACTAC R: CAACCTCTCTGACAGCCAC	MH213271
Catalase (*cat*)	F: GCGTTTGGTTACTTTGAGGTGA R: GAGAAGCGGACAGCAATAGGT	XM_033635388.1
Superoxide dismutase (*sod*)	F: GGAGACAATACAAACGGGTGC R: CCAGCGTTGCCAGTCTTTA	NM001303360.1
Glutathione reductase (*gr)*	F: CTTTCACTCCGATGTATCACGC R: GCTTTGGTAGCACCCATTTTG	XM_033633504.1
MHC class II molecule (*mhc ii*)	F: CAGGTTCAGCAGCAGTTTGG R: AGCAGCCTGGTAGTCAATCCC	JF796053.1
Transforming growth factor-β (*tgf-β*)	F: CGATGTCACTGACGCCCTGC R: AGCCGCGGTCATCACTTATC	GQ205390.1
Interleukin-10 (*il-10*)	F: ACACAGCGCTGCTAGACGAG R: GGGCAGCACCGTGTTCAGAT	KJ741852.1
Interferon-gamma (*ifn-γ*)	F: CCACCAAGATGGAGGCTAAG R: CTGCCACCTCACCATTGCT	JX013936.1
Interleukin-6 (*il-6*)	F: CCGACAGCCCGACAGG R: CTGCTTTTCGTGGCGTTT	JN806222.1
Tumor necrosis factor-α (*tnf-α*)	F: CTGGTGATGTGGAGATGGGTC R: CGTCGTGATGTCTGGCTTTC	HQ011925.1
*β-actin*	F: GGCTACTCCTTCACCACCACA R: TCTGGGCAACGGAACCTCT	AY510710.2

**Table 4:** Effects of dietary steroid saponins on the growth performance of hybrid groupers.

Items	S_0_	S_0.1_	S_0.2_
IBW/g	22.73 ± 0.18	22.62 ± 0.04	22.77 ± 0.10
FBW/g	102.63 ± 2.20 ^b^	102.67 ± 2.51 ^b^	96.52 ± 2.24 ^a^
SR/%	94.67 ± 6.11	96.00 ± 0.00	89.33 ± 2.31
PWG/%	351.50 ± 12.75 ^b^	353.96 ± 11.80 ^b^	323.72 ± 7.37 ^a^
SGR(%/d)	2.69 ± 0.06 ^b^	2.70 ± 0.05 ^b^	2.58 ± 0.03 ^a^
PER	1.77 ± 0.10 ^b^	1.81 ± 0.06 ^b^	1.61 ± 0.02 ^a^
PDR/%	31.57 ± 0.41 ^b^	31.83 ± 1.05 ^b^	27.9 ± 1.36 ^a^
FCR	1.05 ± 0.01	1.02 ± 0.02	1.03 ± 0.01
FR(%BW/d)	2.45 ± 0.15	2.40 ± 0.04	2.58 ± 0.06
CF(g/cm^3^)	2.96 ± 0.07	3.04 ± 0.11	2.82 ± 0.00
HSI/%	3.40 ± 0.03 ^a^	4.68 ± 0.08 ^b^	3.86 ± 0.54 ^ab^
VSI/%	10.31 ± 0.00 ^a^	13.38 ± 0.38 ^b^	10.35 ± 0.28 ^a^

Note: Significant differences (*p* < 0.05) were indicated by different letters. The same as the following tables.

**Table 5:** Effects of dietary steroid saponins on the whole fish composition of hybrid groupers.

Items		S_0_	S_0.1_	S_0.2_
Initial	Moisture	72.70 ± 0.15	72.53 ± 0.02	72.59 ± 0.06
	Crude protein (% DM)	61.15 ± 0.99	60.74 ± 0.13	59.47 ± 0.64
	Crude lipid (% DM)	21.43 ± 0.20	21.44 ± 0.09	21.14 ± 0.16
Final	Moisture	72.43 ± 0.09	73.06 ± 0.43	72.74 ± 1.00
	Crude protein (% DM)	62.66 ± 0.61	62.79 ± 0.33	63.14 ± 0.74
	Crude lipid (% DM)	21.69 ± 0.61	21.56 ± 0.59	21.11 ± 0.46

**Table 6:** Effects of dietary steroid saponins on serum biochemical indexes of hybrid groupers.

Indexes	S_0_	S_0.1_	S_0.2_
TG/(mmol/L)	0.54 ± 0.01 ^b^	0.43 ± 0.02 ^a^	0.66 ± 0.02 ^c^
TC/(mmol/L)	2.00 ± 0.14 ^ab^	1.67 ± 0.14 ^a^	2.13 ± 0.05 ^b^
LDL-C/(mmol/L)	0.25 ± 0.04 ^ab^	0.12 ± 0.13 ^a^	0.36 ± 0.03 ^b^
HDL-C/(mmol/L)	1.11 ± 0.15 ^a^	2.49 ± 0.50 ^b^	1.86 ± 0.20 ^b^
GLU/(mmol/L)	6.31 ± 0.13 ^a^	8.96 ± 0.26 ^b^	8.45 ± 0.55 ^b^

**Table 7:** Effects of dietary steroid saponins on serum antioxidative indexes of hybrid groupers.

Indexes	S_0_	S_0.1_	S_0.2_
MDA/(nmol/mL)	4.44 ± 0.22 ^b^	3.24 ± 0.11 ^a^	4.24 ± 0.21 ^b^
SOD/(U/mL)	309.52 ± 8.19 ^b^	427.55 ± 18.60 ^c^	251.81 ± 33.39 ^a^
CAT/(U/mL)	5.42 ± 0.31 ^a^	7.70 ± 0.56 ^b^	7.15 ± 0.89 ^b^
GSH-PX/(U/L)	266.52 ± 3.77 ^a^	285.00 ± 6.46 ^b^	274.57 ± 6.46 ^a^

## Data Availability

The data presented in this study are available in the main article.
